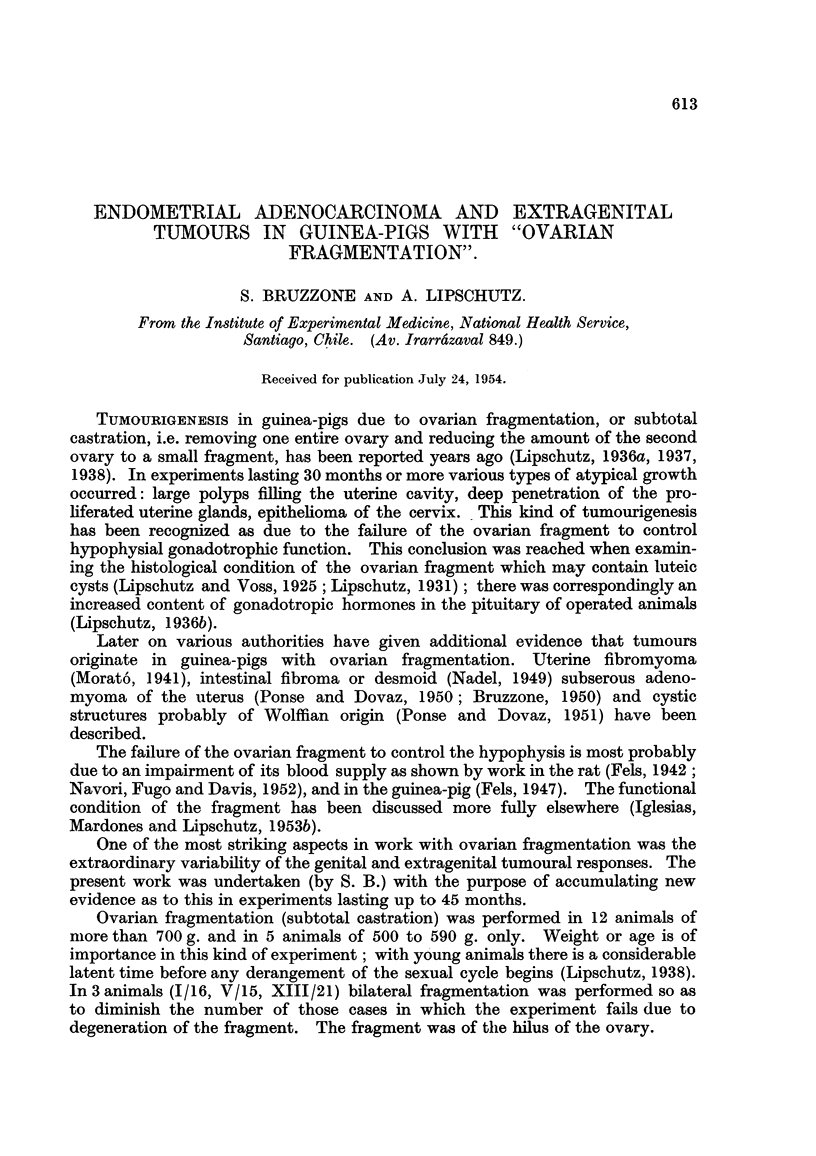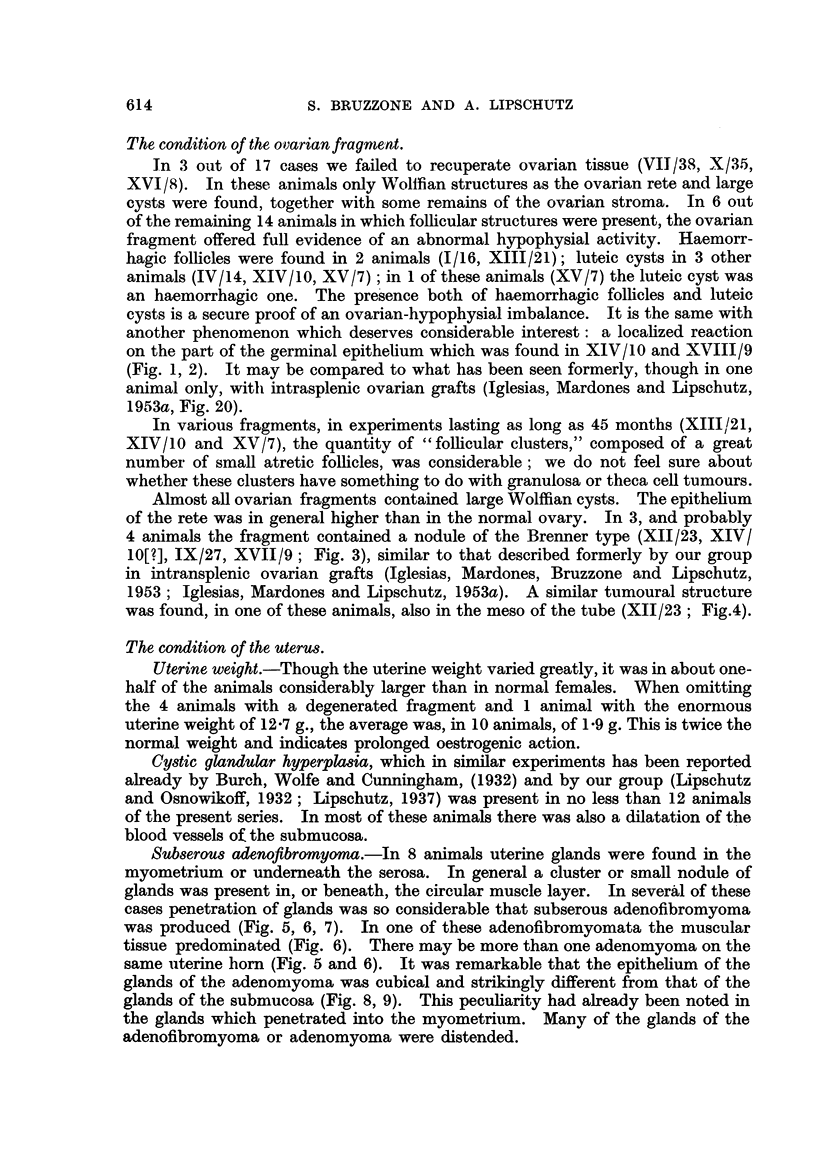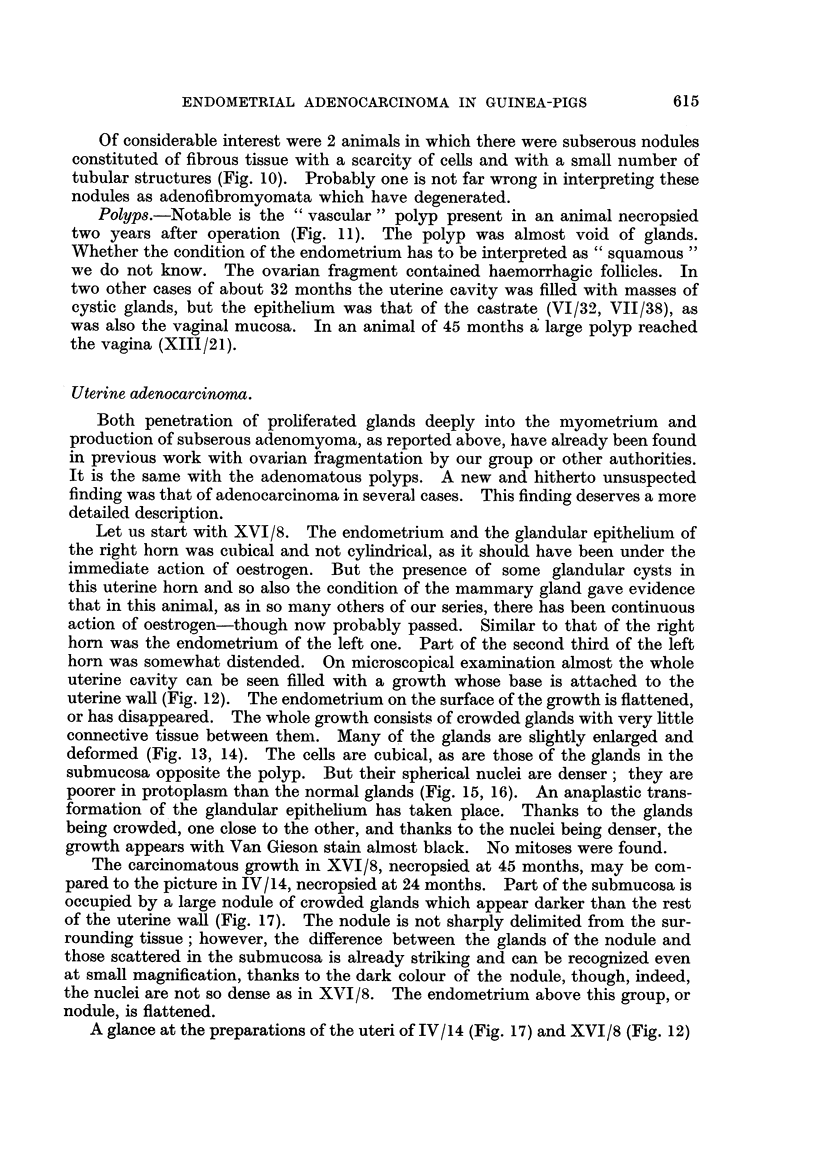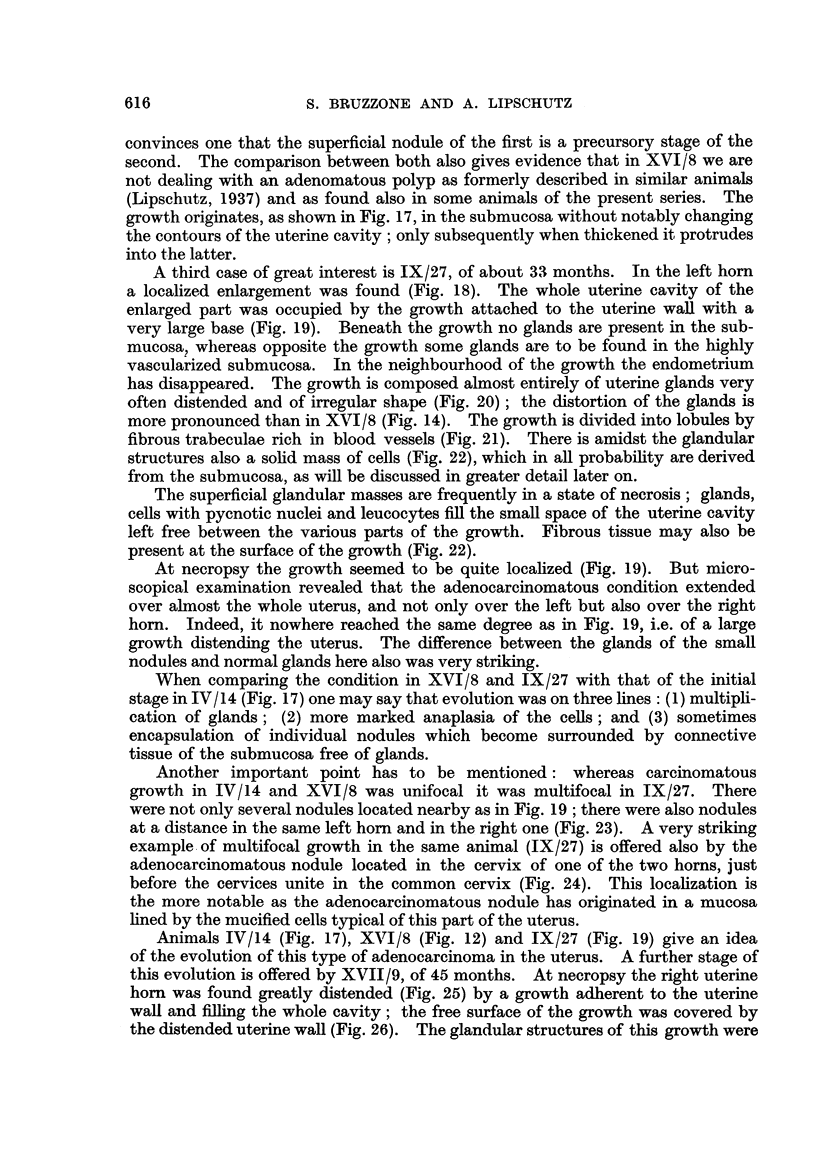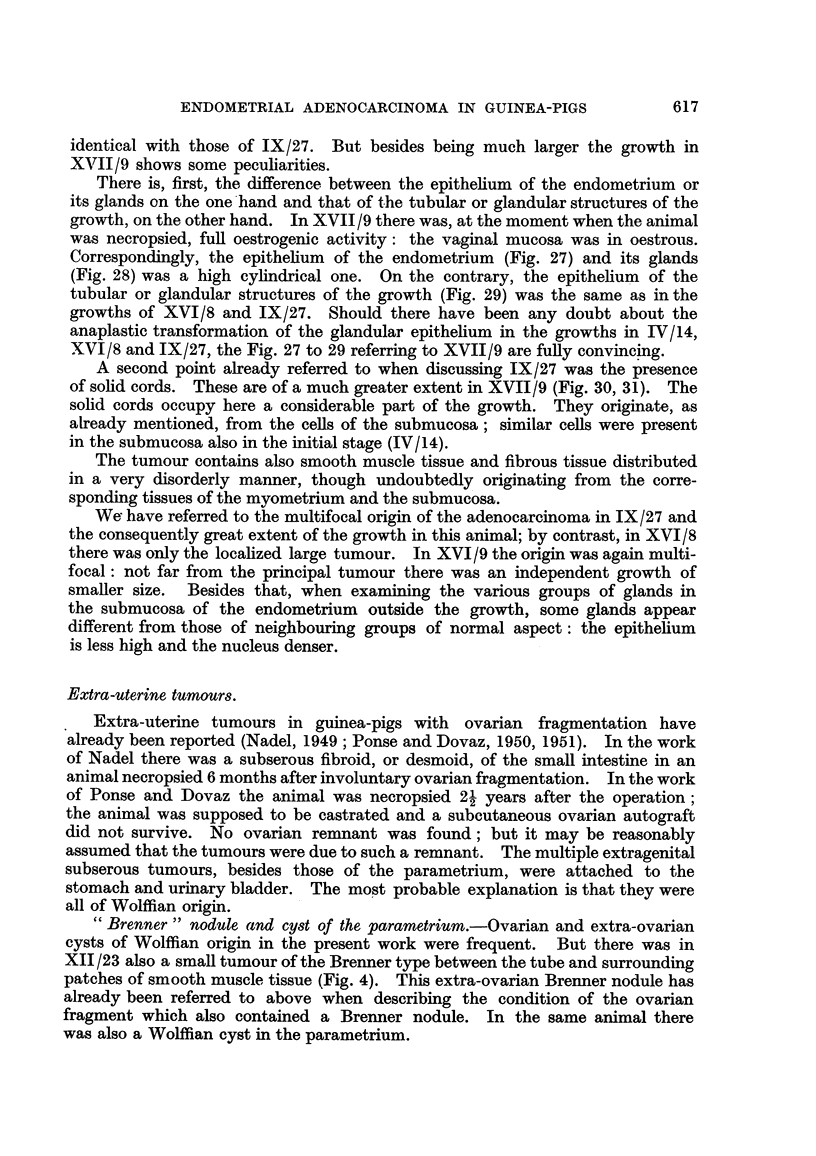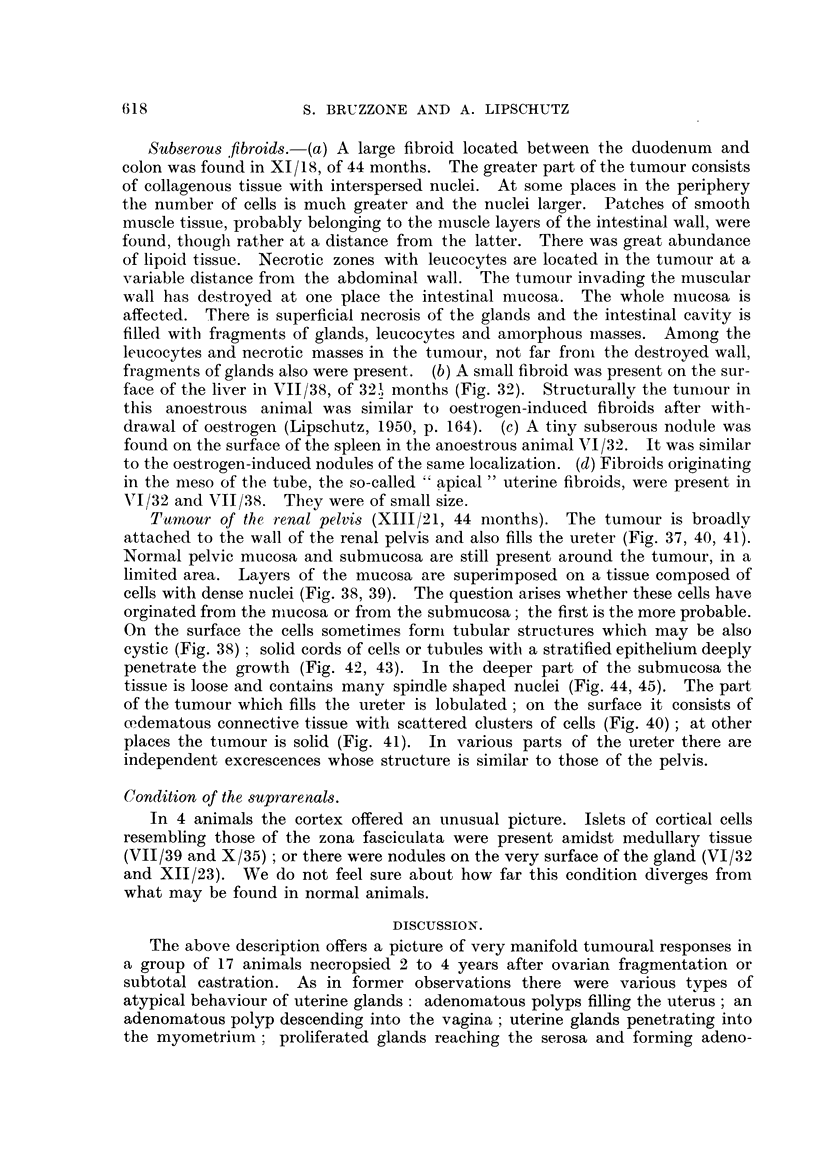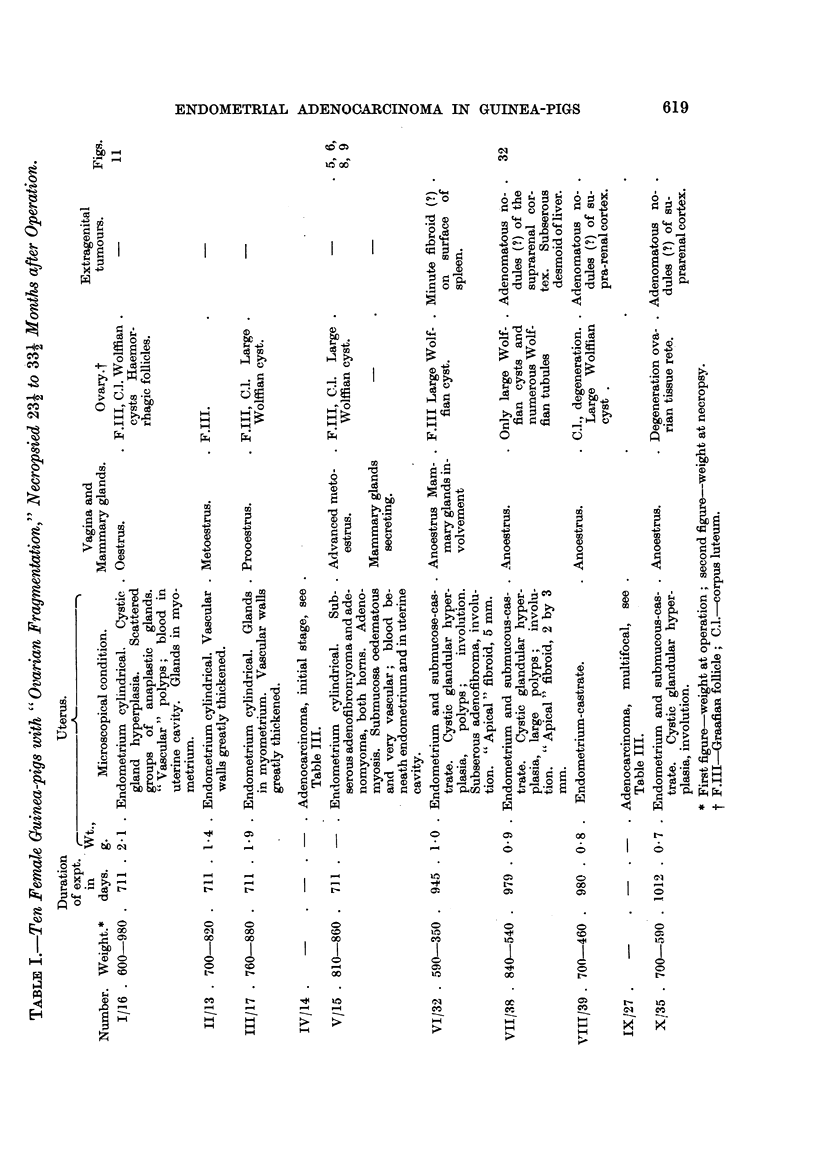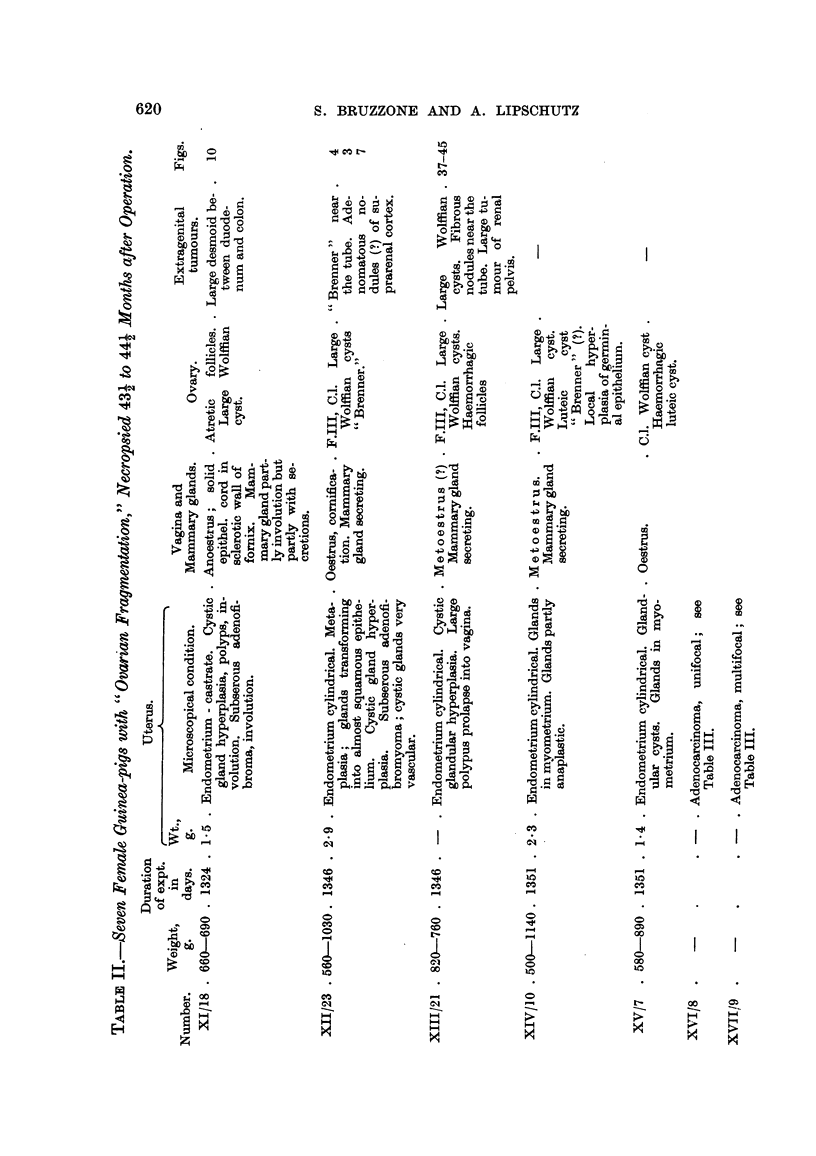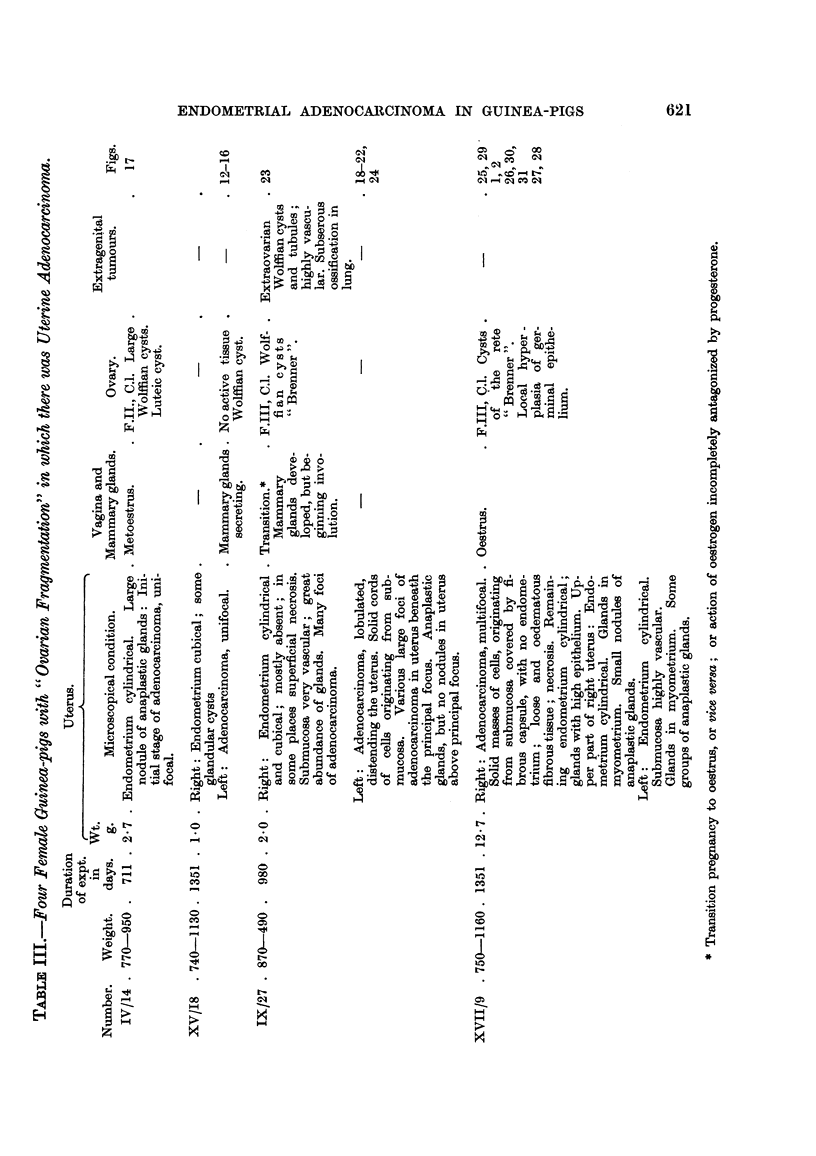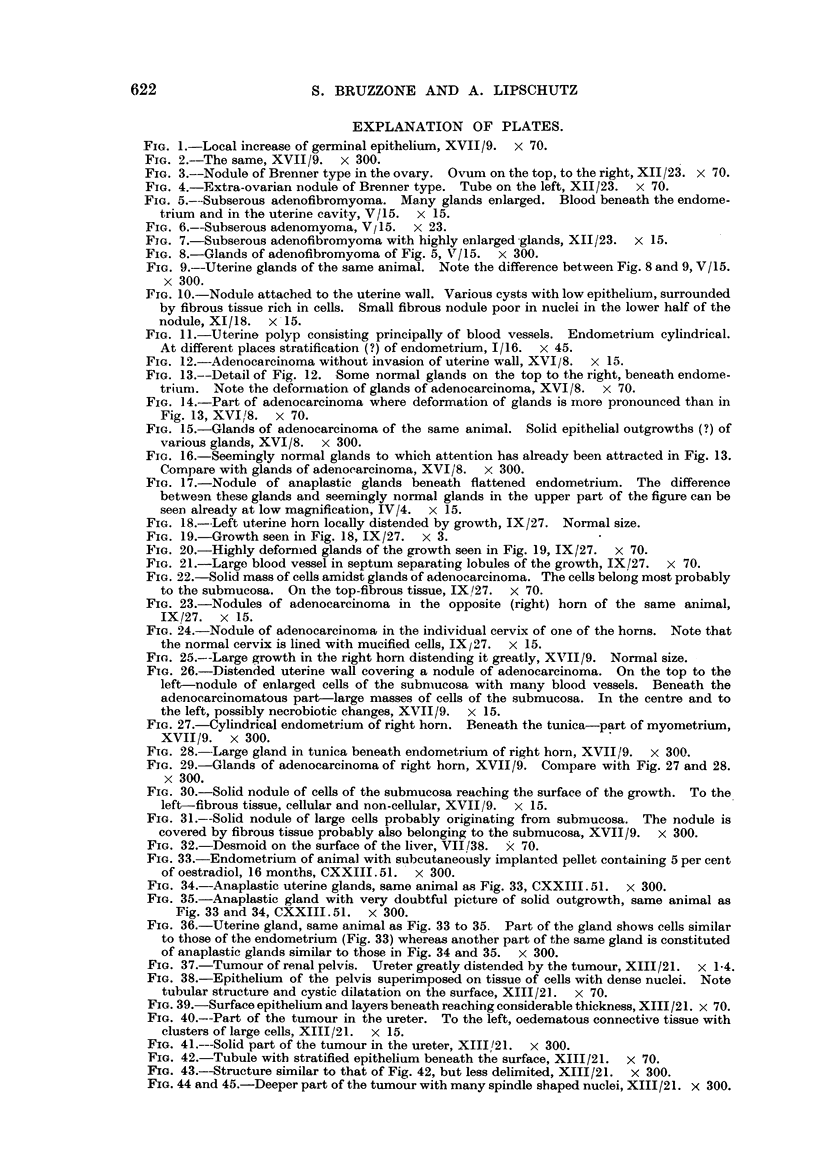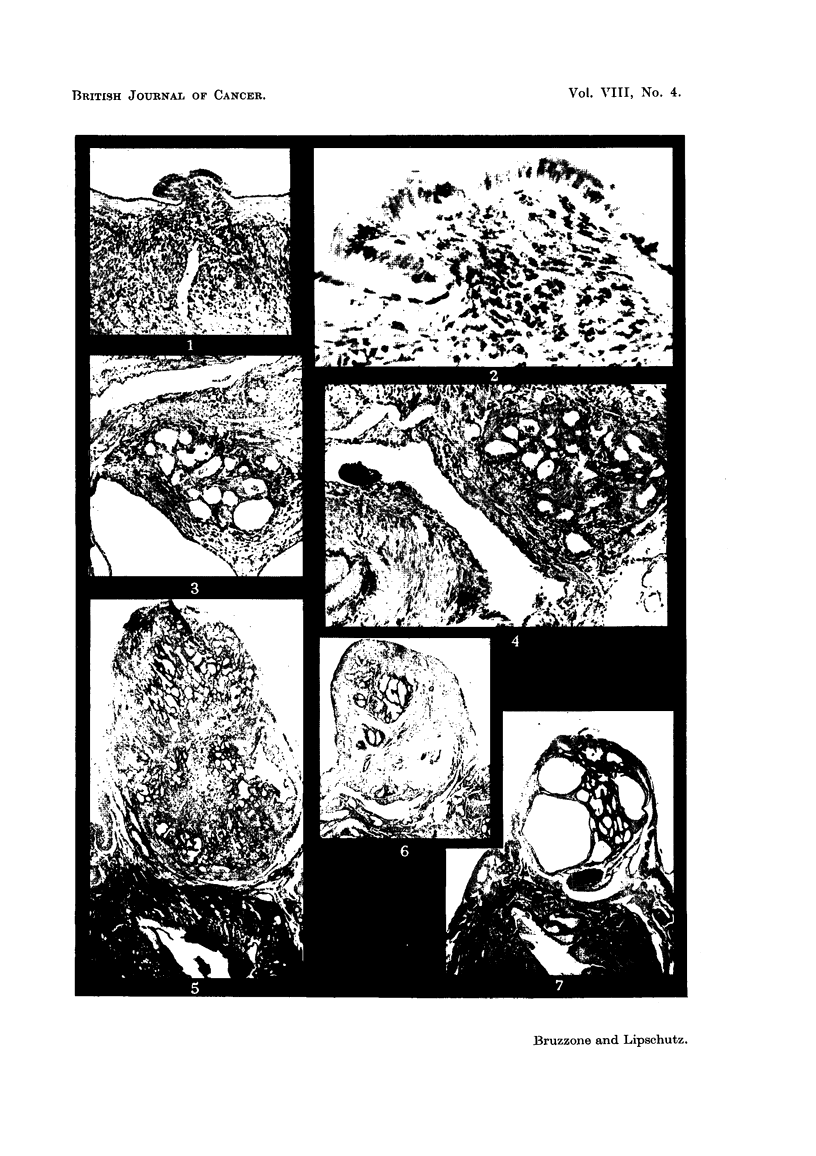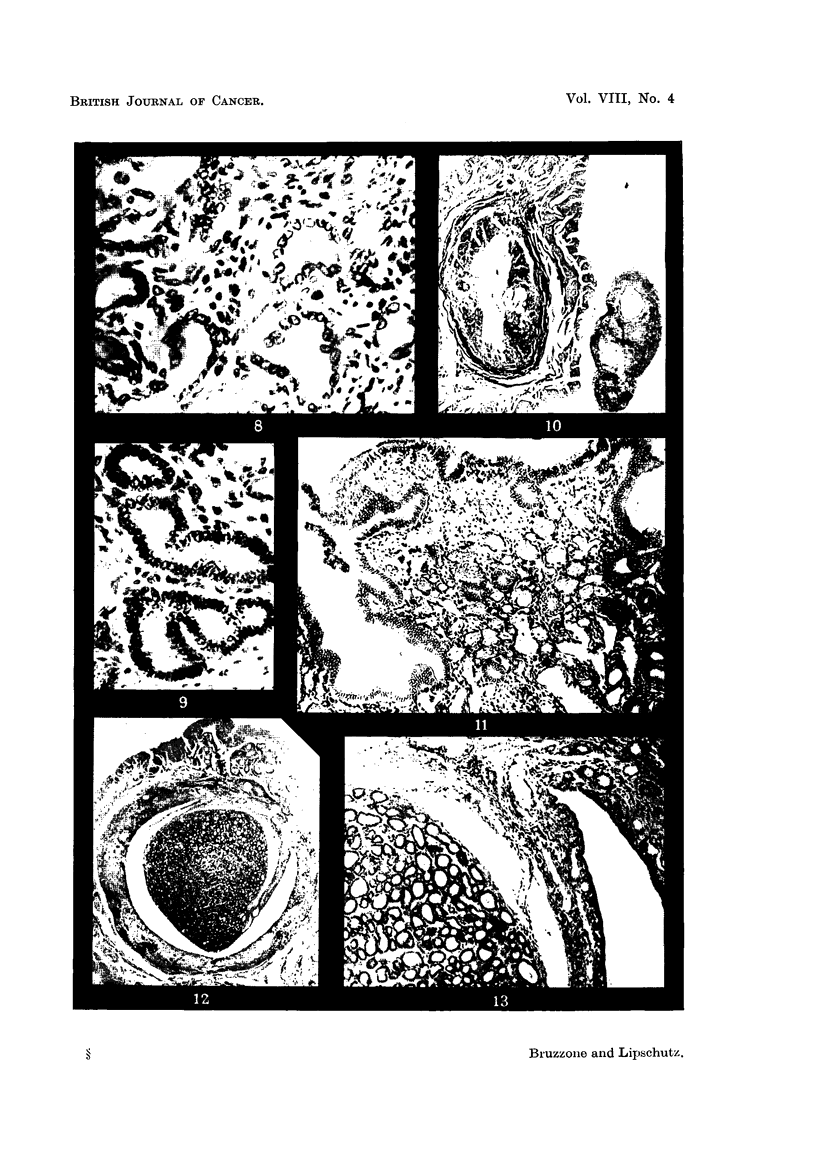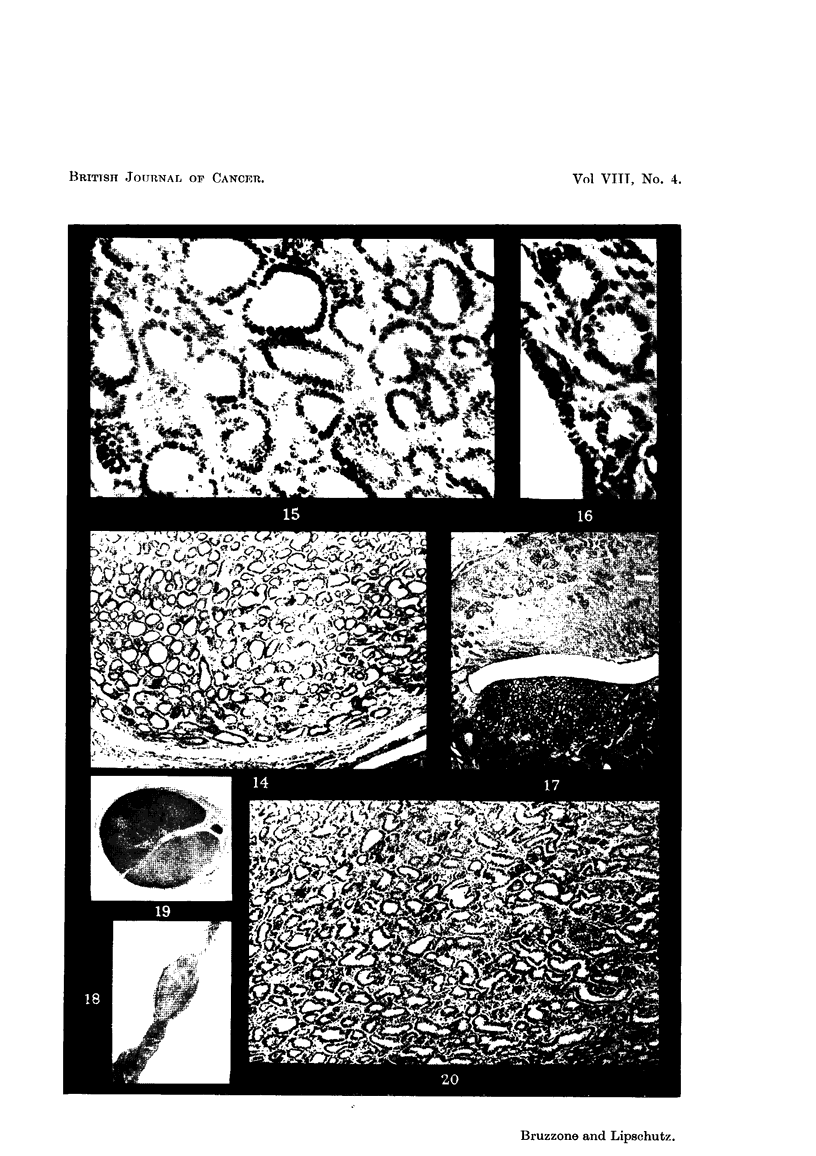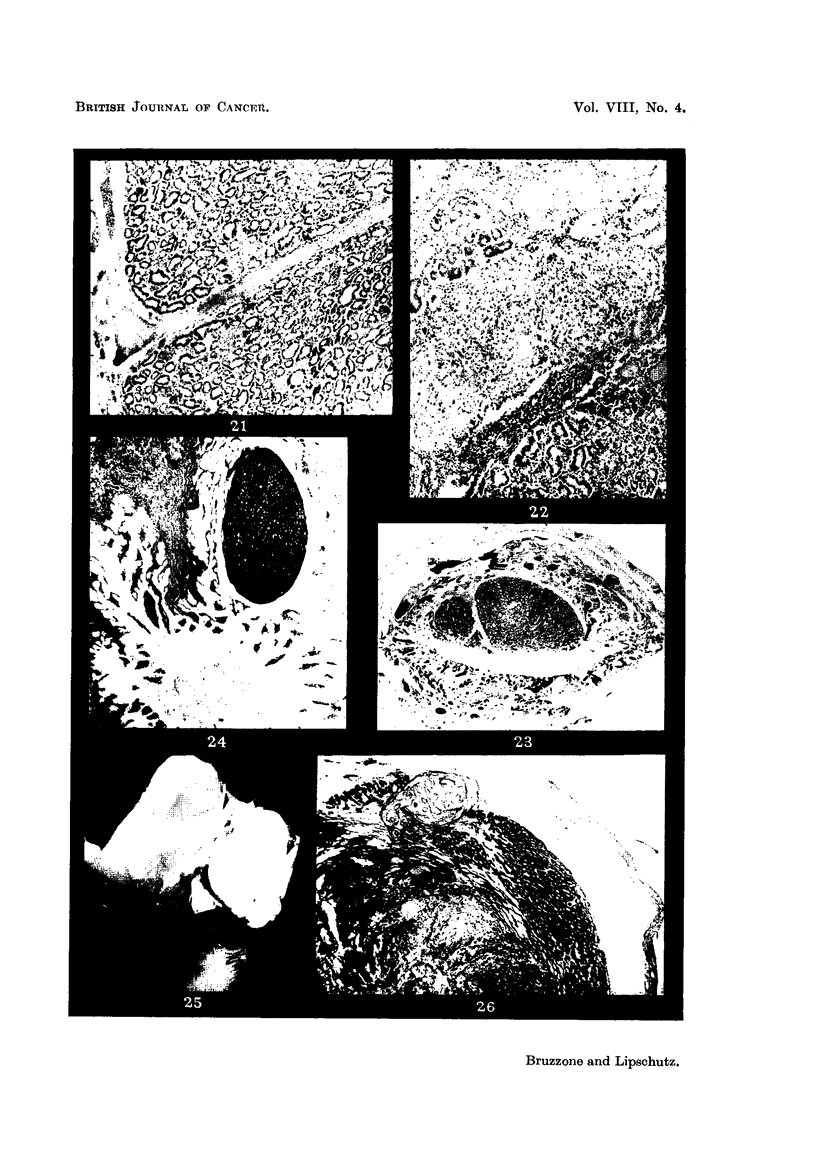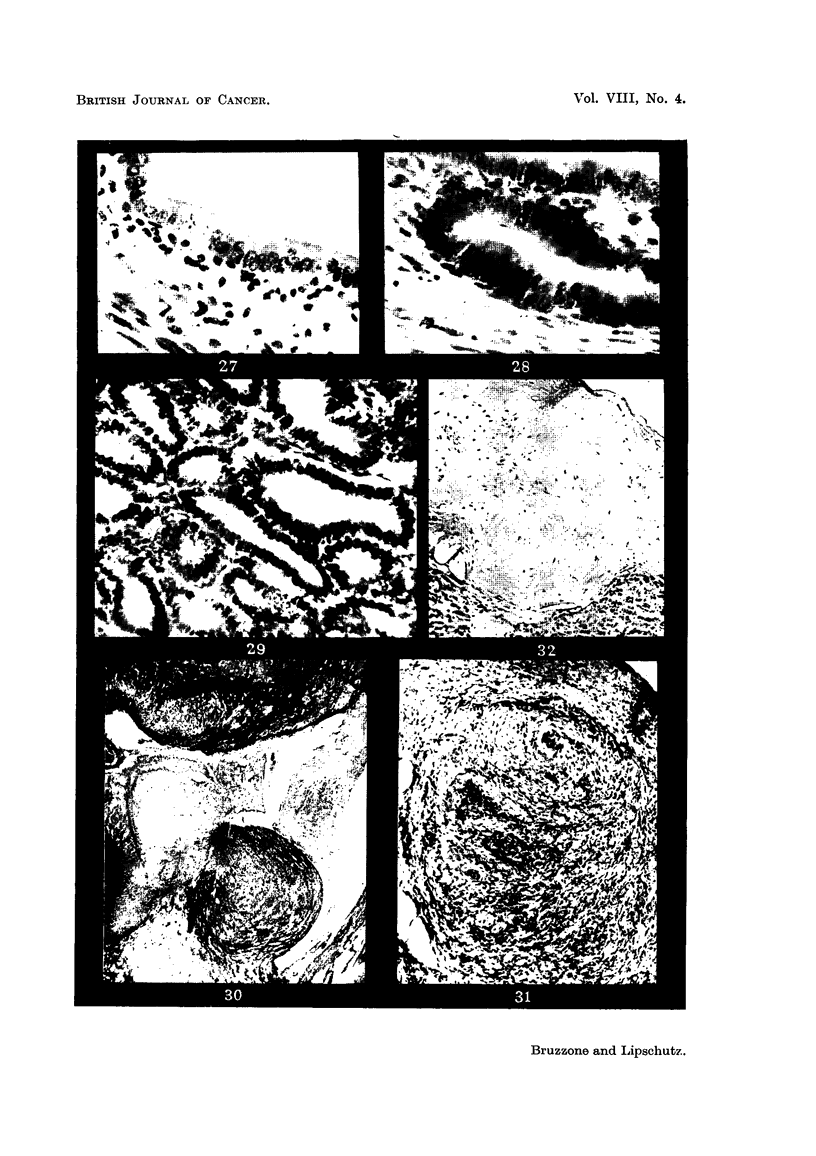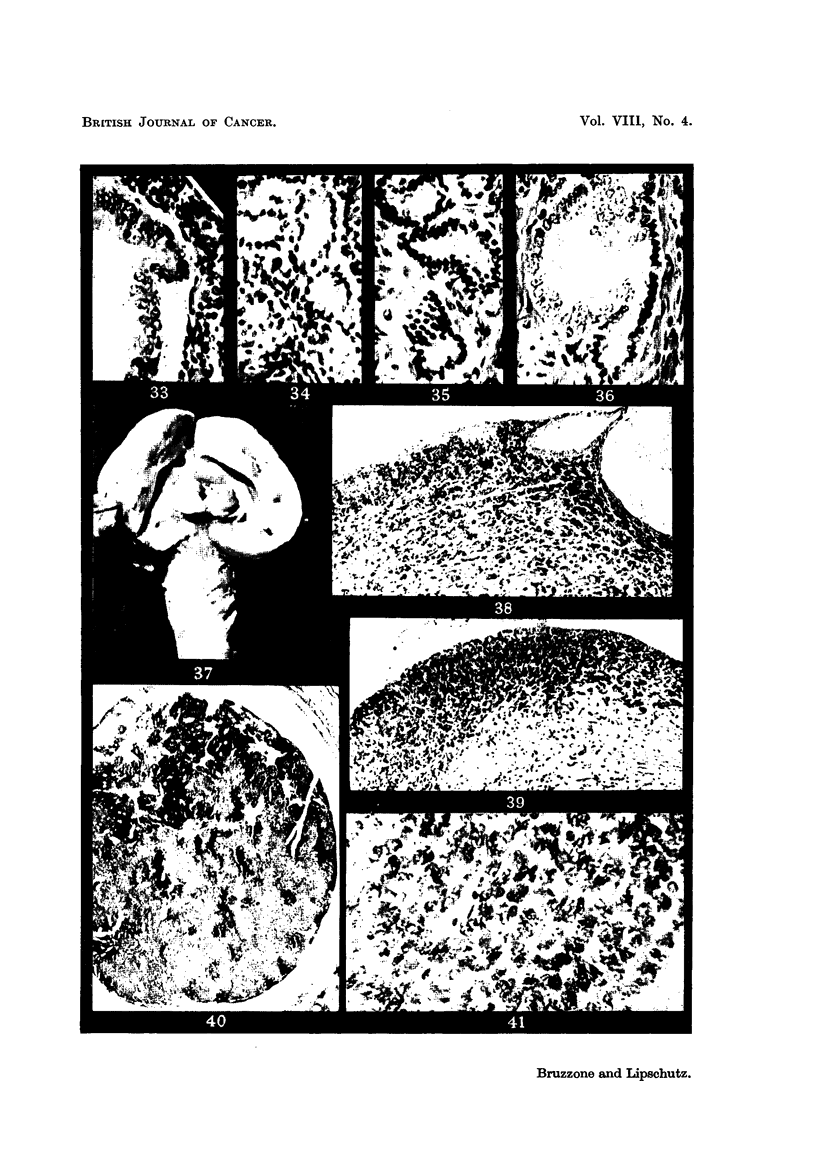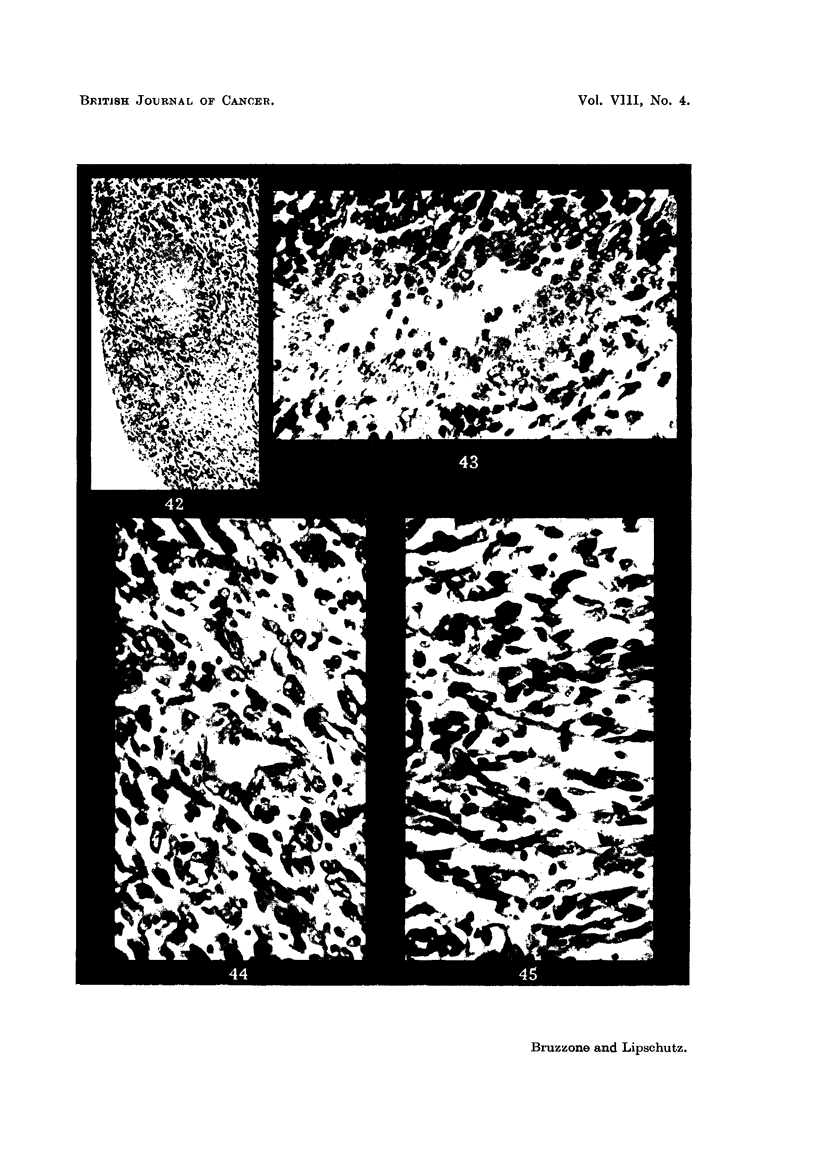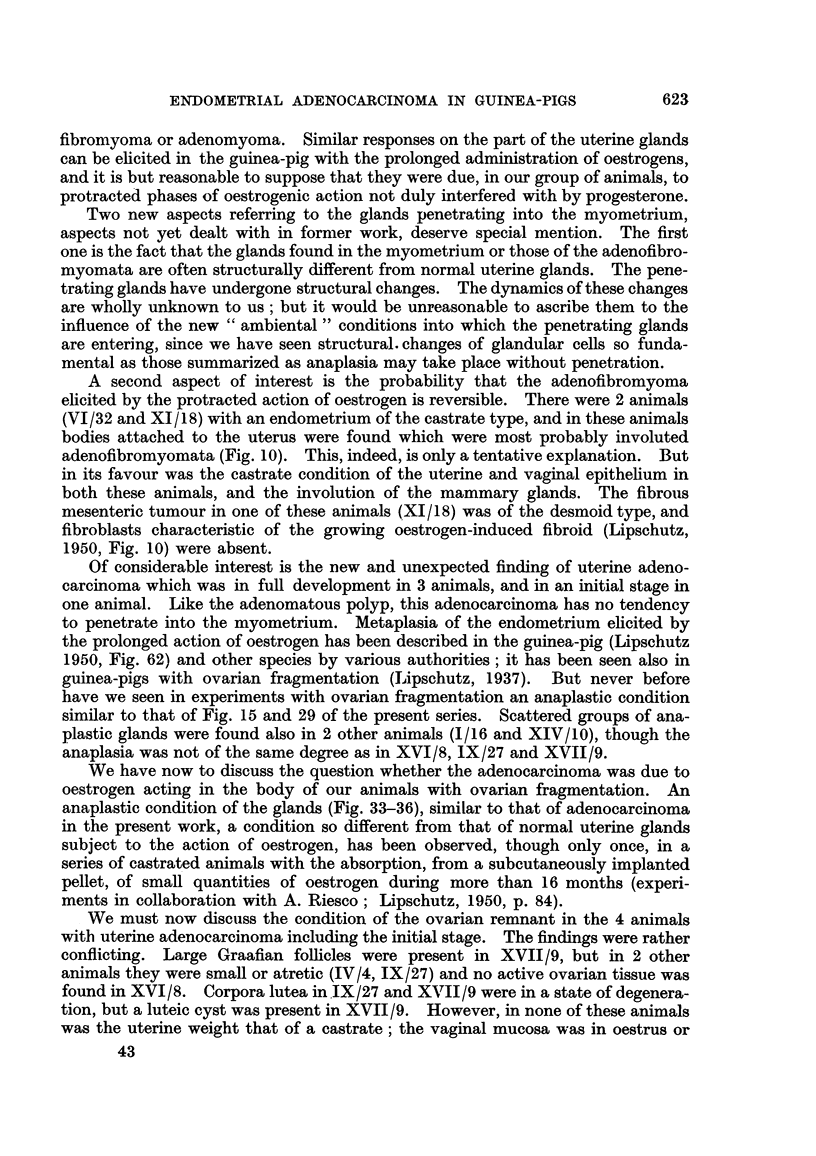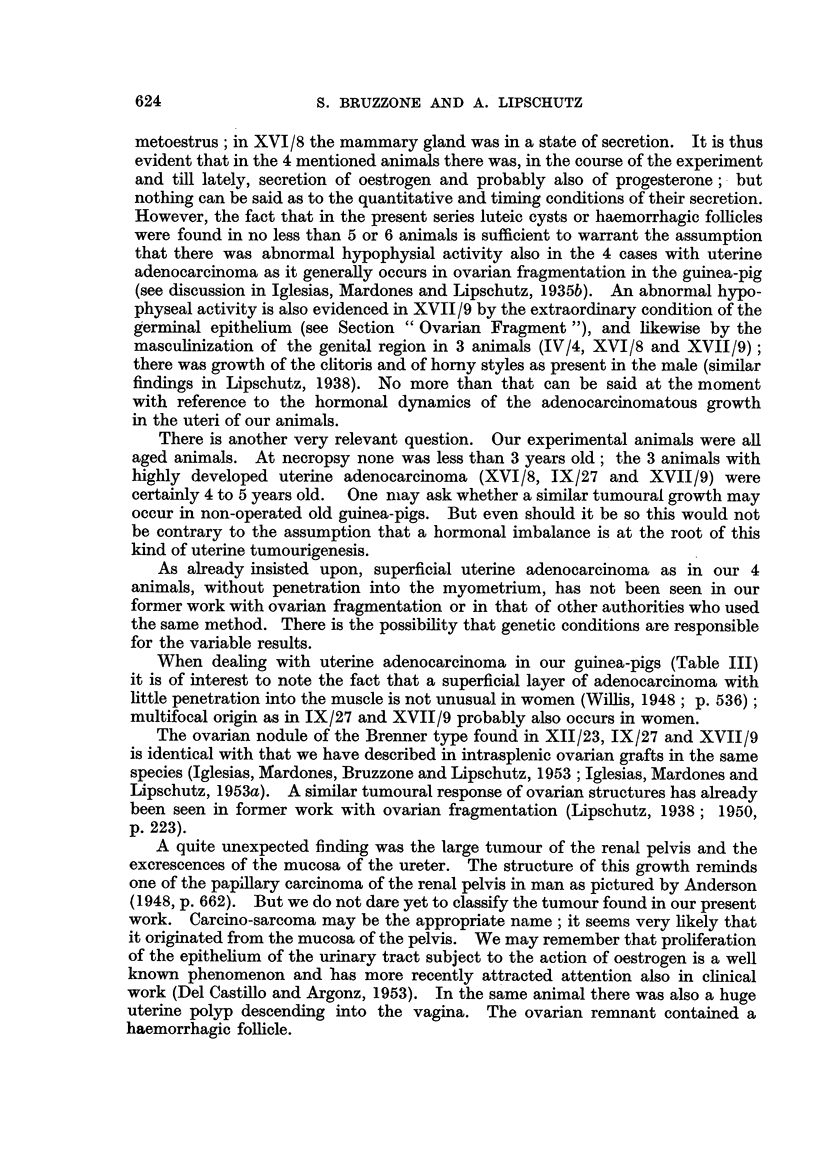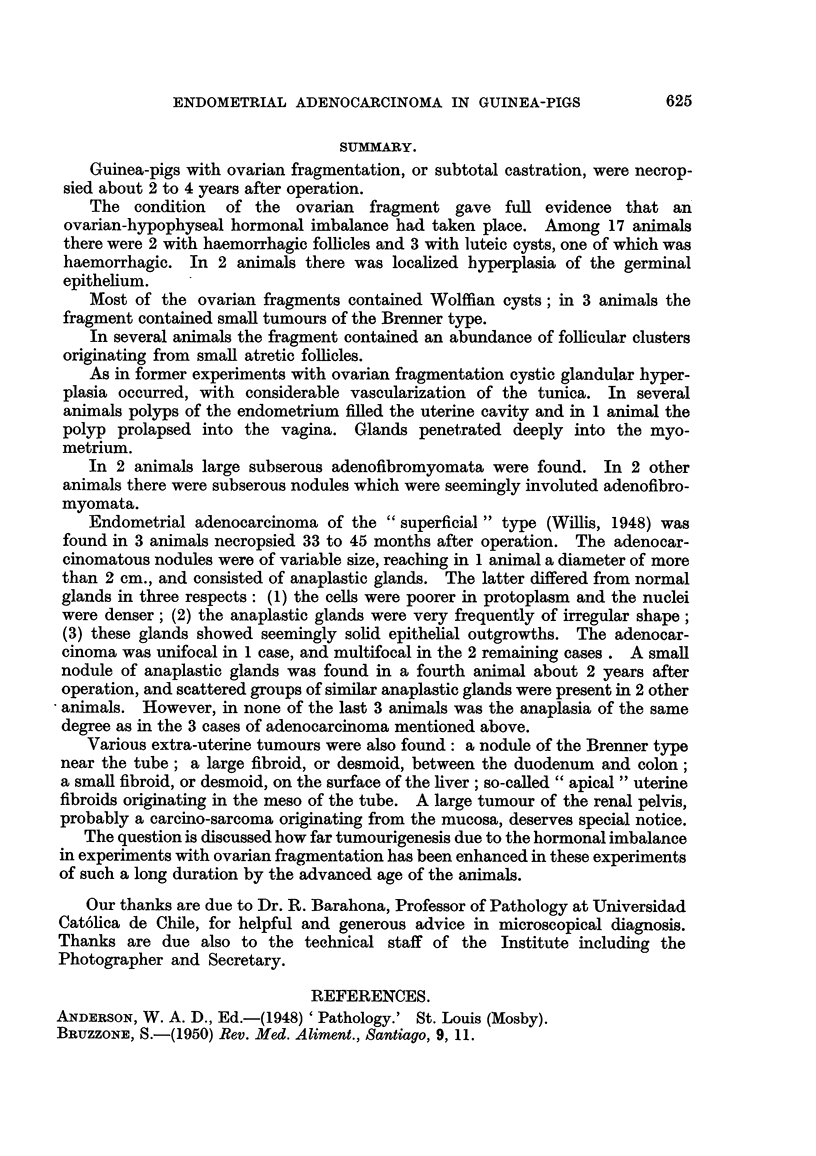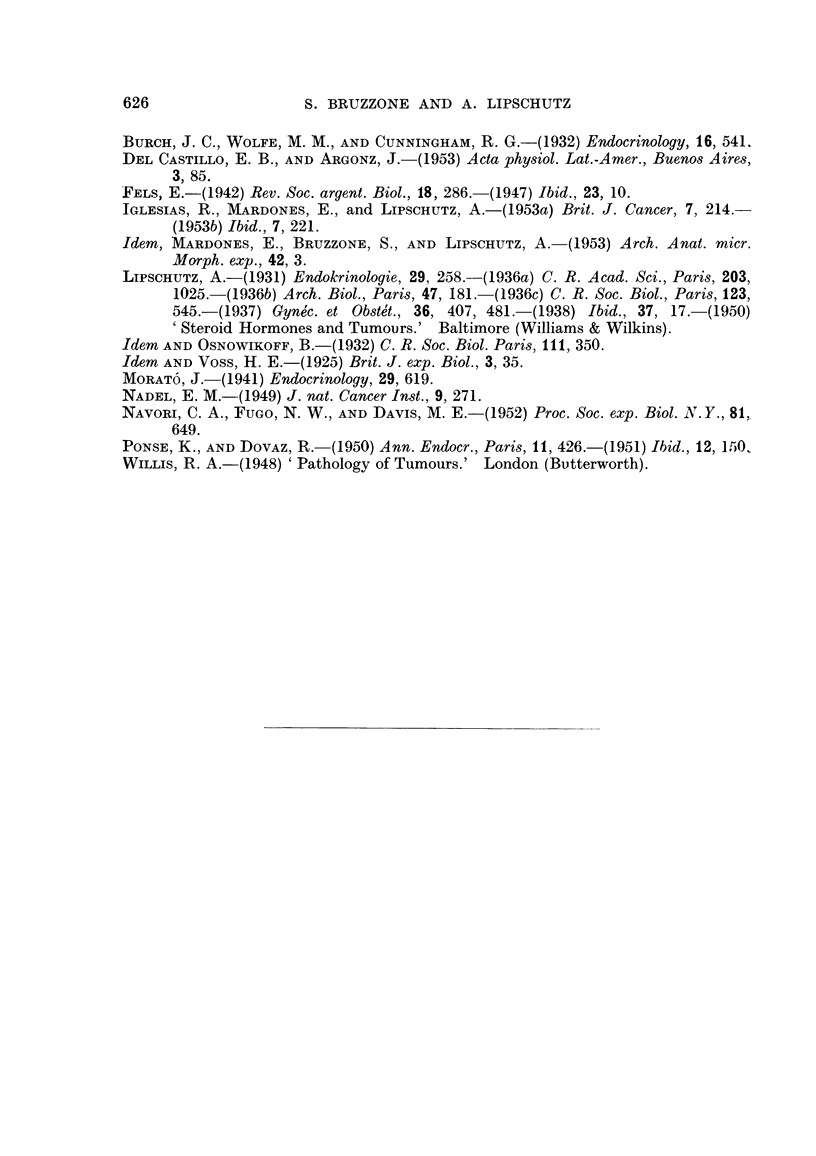# Endometrial Adenocarcinoma and Extragenital Tumours in Guinea-pigs with “Ovarian Fragmentation”

**DOI:** 10.1038/bjc.1954.67

**Published:** 1954-12

**Authors:** S. Bruzzone, A. Lipschutz

## Abstract

**Images:**


### ENDOMETRIAL ADENOCARCINOMA AND EXTRAGENITAL TUMOURS IN GUINEA-PIGS WITH "OVARIAN FRAGMENTATION".

S. BRUZZONE AND A. LIPSCHUTZ.

*From the Institute of Experimental Medicine, National Health Service, Santiago, Chile. (Av. Irarrázaval 849.)*

Received for publication July 24, 1954.

TUMOURIGENESIS in guinea-pigs due to ovarian fragmentation, or subtotal castration, i.e. removing one entire ovary and reducing the amount of the second ovary to a small fragment, has been reported years ago (Lipschutz, 1936*a*, 1937, 1938). In experiments lasting 30 months or more various types of atypical growth occurred: large polyps filling the uterine cavity, deep penetration of the proliferated uterine glands, epithelioma of the cervix. This kind of tumourigenesis has been recognized as due to the failure of the ovarian fragment to control hypophysial gonadotrophic function. This conclusion was reached when examining the histological condition of the ovarian fragment which may contain luteic cysts (Lipschutz and Voss, 1925 ; Lipschutz, 1931) ; there was correspondingly an increased content of gonadotropic hormones in the pituitary of operated animals (Lipschutz, 1936*b*).

Later on various authorities have given additional evidence that tumours originate in guinea-pigs with ovarian fragmentation. Uterine fibromyoma (Morató, 1941), intestinal fibroma or desmoid (Nadel, 1949) subserous adenomyoma of the uterus (Ponse and Dovaz, 1950 ; Bruzzone, 1950) and cystic structures probably of Wolffian origin (Ponse and Dovaz, 1951) have been described.

The failure of the ovarian fragment to control the hypophysis is most probably due to an impairment of its blood supply as shown by work in the rat (Fels, 1942 ; Navori, Fugo and Davis, 1952), and in the guinea-pig (Fels, 1947). The functional condition of the fragment has been discussed more fully elsewhere (Iglesias, Mardones and Lipschutz, 1953*b*).

One of the most striking aspects in work with ovarian fragmentation was the extraordinary variability of the genital and extragenital tumoural responses. The present work was undertaken (by S. B.) with the purpose of accumulating new evidence as to this in experiments lasting up to 45 months.

Ovarian fragmentation (subtotal castration) was performed in 12 animals of more than 700 g. and in 5 animals of 500 to 590 g. only. Weight or age is of importance in this kind of experiment ; with young animals there is a considerable latent time before any derangement of the sexual cycle begins (Lipschutz, 1938). In 3 animals (I/16, V/15, XIII/21) bilateral fragmentation was performed so as to diminish the number of those cases in which the experiment fails due to degeneration of the fragment. The fragment was of the hilus of the ovary.

*The condition of the ovarian fragment.*

In 3 out of 17 cases we failed to recuperate ovarian tissue (VII/38, X/35, XVI/8). In these animals only Wolffian structures as the ovarian rete and large cysts were found, together with some remains of the ovarian stroma. In 6 out of the remaining 14 animals in which follicular structures were present, the ovarian fragment offered full evidence of an abnormal hypophysial activity. Haemorrhagic follicles were found in 2 animals (I/16, XIII/21); luteic cysts in 3 other animals (IV/14, XIV/10, XV/7); in 1 of these animals (XV/7) the luteic cyst was an haemorrhagic one. The presence both of haemorrhagic follicles and luteic cysts is a secure proof of an ovarian-hypophysial imbalance. It is the same with another phenomenon which deserves considerable interest: a localized reaction on the part of the germinal epithelium which was found in XIV/10 and XVIII/9 (Fig. 1, 2). It may be compared to what has been seen formerly, though in one animal only, with intrasplenic ovarian grafts (Iglesias, Mardones and Lipschutz, 1953*a*, Fig. 20).

In various fragments, in experiments lasting as long as 45 months (XIII/21, XIV/10 and XV/7), the quantity of "follicular clusters," composed of a great number of small atretic follicles, was considerable; we do not feel sure about whether these clusters have something to do with granulosa or theca cell tumours.

Almost all ovarian fragments contained large Wolffian cysts. The epithelium of the rete was in general higher than in the normal ovary. In 3, and probably 4 animals the fragment contained a nodule of the Brenner type (XII/23, XIV/10[?], IX/27, XVII/9; Fig. 3), similar to that described formerly by our group in intransplenic ovarian grafts (Iglesias, Mardones, Bruzzone and Lipschutz, 1953; Iglesias, Mardones and Lipschutz, 1953*a*). A similar tumoural structure was found, in one of these animals, also in the meso of the tube (XII/23; Fig.4).

*The condition of the uterus.*

*Uterine weight.*—Though the uterine weight varied greatly, it was in about one-half of the animals considerably larger than in normal females. When omitting the 4 animals with a degenerated fragment and 1 animal with the enormous uterine weight of 12·7 g., the average was, in 10 animals, of 1·9 g. This is twice the normal weight and indicates prolonged oestrogenic action.

*Cystic glandular hyperplasia*, which in similar experiments has been reported already by Burch, Wolfe and Cunningham, (1932) and by our group (Lipschutz and Osnowikoff, 1932; Lipschutz, 1937) was present in no less than 12 animals of the present series. In most of these animals there was also a dilatation of the blood vessels of the submucosa.

*Subserous adenofibromyoma.*—In 8 animals uterine glands were found in the myometrium or underneath the serosa. In general a cluster or small nodule of glands was present in, or beneath, the circular muscle layer. In several of these cases penetration of glands was so considerable that subserous adenofibromyoma was produced (Fig. 5, 6, 7). In one of these adenofibromyomata the muscular tissue predominated (Fig. 6). There may be more than one adenomyoma on the same uterine horn (Fig. 5 and 6). It was remarkable that the epithelium of the glands of the adenomyoma was cubical and strikingly different from that of the glands of the submucosa (Fig. 8, 9). This peculiarity had already been noted in the glands which penetrated into the myometrium. Many of the glands of the adenofibromyoma or adenomyoma were distended.

Of considerable interest were 2 animals in which there were subserous nodules constituted of fibrous tissue with a scarcity of cells and with a small number of tubular structures (Fig. 10). Probably one is not far wrong in interpreting these nodules as adenofibromyomata which have degenerated.

*Polyps.*—Notable is the " vascular " polyp present in an animal necropsied two years after operation (Fig. 11). The polyp was almost void of glands. Whether the condition of the endometrium has to be interpreted as " squamous " we do not know. The ovarian fragment contained haemorrhagic follicles. In two other cases of about 32 months the uterine cavity was filled with masses of cystic glands, but the epithelium was that of the castrate (VI/32, VII/38), as was also the vaginal mucosa. In an animal of 45 months a large polyp reached the vagina (XIII/21).

*Uterine adenocarcinoma.*

Both penetration of proliferated glands deeply into the myometrium and production of subserous adenomyoma, as reported above, have already been found in previous work with ovarian fragmentation by our group or other authorities. It is the same with the adenomatous polyps. A new and hitherto unsuspected finding was that of adenocarcinoma in several cases. This finding deserves a more detailed description.

Let us start with XVI/8. The endometrium and the glandular epithelium of the right horn was cubical and not cylindrical, as it should have been under the immediate action of oestrogen. But the presence of some glandular cysts in this uterine horn and so also the condition of the mammary gland gave evidence that in this animal, as in so many others of our series, there has been continuous action of oestrogen—though now probably passed. Similar to that of the right horn was the endometrium of the left one. Part of the second third of the left horn was somewhat distended. On microscopical examination almost the whole uterine cavity can be seen filled with a growth whose base is attached to the uterine wall (Fig. 12). The endometrium on the surface of the growth is flattened, or has disappeared. The whole growth consists of crowded glands with very little connective tissue between them. Many of the glands are slightly enlarged and deformed (Fig. 13, 14). The cells are cubical, as are those of the glands in the submucosa opposite the polyp. But their spherical nuclei are denser ; they are poorer in protoplasm than the normal glands (Fig. 15, 16). An anaplastic transformation of the glandular epithelium has taken place. Thanks to the glands being crowded, one close to the other, and thanks to the nuclei being denser, the growth appears with Van Gieson stain almost black. No mitoses were found.

The carcinomatous growth in XVI/8, necropsied at 45 months, may be compared to the picture in IV/14, necropsied at 24 months. Part of the submucosa is occupied by a large nodule of crowded glands which appear darker than the rest of the uterine wall (Fig. 17). The nodule is not sharply delimited from the surrounding tissue ; however, the difference between the glands of the nodule and those scattered in the submucosa is already striking and can be recognized even at small magnification, thanks to the dark colour of the nodule, though, indeed, the nuclei are not so dense as in XVI/8. The endometrium above this group, or nodule, is flattened.

A glance at the preparations of the uteri of IV/14 (Fig. 17) and XVI/8 (Fig. 12)

convinces one that the superficial nodule of the first is a precursory stage of the second. The comparison between both also gives evidence that in XVI/8 we are not dealing with an adenomatous polyp as formerly described in similar animals (Lipschutz, 1937) and as found also in some animals of the present series. The growth originates, as shown in Fig. 17, in the submucosa without notably changing the contours of the uterine cavity ; only subsequently when thickened it protrudes into the latter.

A third case of great interest is IX/27, of about 33 months. In the left horn a localized enlargement was found (Fig. 18). The whole uterine cavity of the enlarged part was occupied by the growth attached to the uterine wall with a very large base (Fig. 19). Beneath the growth no glands are present in the submucosa, whereas opposite the growth some glands are to be found in the highly vascularized submucosa. In the neighbourhood of the growth the endometrium has disappeared. The growth is composed almost entirely of uterine glands very often distended and of irregular shape (Fig. 20) ; the distortion of the glands is more pronounced than in XVI/8 (Fig. 14). The growth is divided into lobules by fibrous trabeculae rich in blood vessels (Fig. 21). There is amidst the glandular structures also a solid mass of cells (Fig. 22), which in all probability are derived from the submucosa, as will be discussed in greater detail later on.

The superficial glandular masses are frequently in a state of necrosis ; glands, cells with pycnotic nuclei and leucocytes fill the small space of the uterine cavity left free between the various parts of the growth. Fibrous tissue may also be present at the surface of the growth (Fig. 22).

At necropsy the growth seemed to be quite localized (Fig. 19). But microscopical examination revealed that the adenocarcinomatous condition extended over almost the whole uterus, and not only over the left but also over the right horn. Indeed, it nowhere reached the same degree as in Fig. 19, i.e. of a large growth distending the uterus. The difference between the glands of the small nodules and normal glands here also was very striking.

When comparing the condition in XVI/8 and IX/27 with that of the initial stage in IV/14 (Fig. 17) one may say that evolution was on three lines : (1) multiplication of glands ; (2) more marked anaplasia of the cells ; and (3) sometimes encapsulation of individual nodules which become surrounded by connective tissue of the submucosa free of glands.

Another important point has to be mentioned : whereas carcinomatous growth in IV/14 and XVI/8 was unifocal it was multifocal in IX/27. There were not only several nodules located nearby as in Fig. 19 ; there were also nodules at a distance in the same left horn and in the right one (Fig. 23). A very striking example of multifocal growth in the same animal (IX/27) is offered also by the adenocarcinomatous nodule located in the cervix of one of the two horns, just before the cervices unite in the common cervix (Fig. 24). This localization is the more notable as the adenocarcinomatous nodule has originated in a mucosa lined by the mucified cells typical of this part of the uterus.

Animals IV/14 (Fig. 17), XVI/8 (Fig. 12) and IX/27 (Fig. 19) give an idea of the evolution of this type of adenocarcinoma in the uterus. A further stage of this evolution is offered by XVII/9, of 45 months. At necropsy the right uterine horn was found greatly distended (Fig. 25) by a growth adherent to the uterine wall and filling the whole cavity ; the free surface of the growth was covered by the distended uterine wall (Fig. 26). The glandular structures of this growth were identical with those of IX/27. But besides being much larger the growth in XVII/9 shows some peculiarities.

There is, first, the difference between the epithelium of the endometrium or its glands on the one hand and that of the tubular or glandular structures of the growth, on the other hand. In XVII/9 there was, at the moment when the animal was necropsied, full oestrogenic activity : the vaginal mucosa was in oestrous. Correspondingly, the epithelium of the endometrium (Fig. 27) and its glands (Fig. 28) was a high cylindrical one. On the contrary, the epithelium of the tubular or glandular structures of the growth (Fig. 29) was the same as in the growths of XVI/8 and IX/27. Should there have been any doubt about the anaplastic transformation of the glandular epithelium in the growths in IV/14, XVI/8 and IX/27, the Fig. 27 to 29 referring to XVII/9 are fully convincing.

A second point already referred to when discussing IX/27 was the presence of solid cords. These are of a much greater extent in XVII/9 (Fig. 30, 31). The solid cords occupy here a considerable part of the growth. They originate, as already mentioned, from the cells of the submucosa ; similar cells were present in the submucosa also in the initial stage (IV/14).

The tumour contains also smooth muscle tissue and fibrous tissue distributed in a very disorderly manner, though undoubtedly originating from the corresponding tissues of the myometrium and the submucosa.

We have referred to the multifocal origin of the adenocarcinoma in IX/27 and the consequently great extent of the growth in this animal; by contrast, in XVI/8 there was only the localized large tumour. In XVI/9 the origin was again multifocal : not far from the principal tumour there was an independent growth of smaller size. Besides that, when examining the various groups of glands in the submucosa of the endometrium outside the growth, some glands appear different from those of neighbouring groups of normal aspect : the epithelium is less high and the nucleus denser.

*Extra-uterine tumours.*

Extra-uterine tumours in guinea-pigs with ovarian fragmentation have already been reported (Nadel, 1949 ; Ponse and Dovaz, 1950, 1951). In the work of Nadel there was a subserous fibroid, or desmoid, of the small intestine in an animal necropsied 6 months after involuntary ovarian fragmentation. In the work of Ponse and Dovaz the animal was necropsied $2\frac{1}{2}$ years after the operation ; the animal was supposed to be castrated and a subcutaneous ovarian autograft did not survive. No ovarian remnant was found ; but it may be reasonably assumed that the tumours were due to such a remnant. The multiple extragenital subserous tumours, besides those of the parametrium, were attached to the stomach and urinary bladder. The most probable explanation is that they were all of Wolffian origin.

*" Brenner " nodule and cyst of the parametrium.*—Ovarian and extra-ovarian cysts of Wolffian origin in the present work were frequent. But there was in XII/23 also a small tumour of the Brenner type between the tube and surrounding patches of smooth muscle tissue (Fig. 4). This extra-ovarian Brenner nodule has already been referred to above when describing the condition of the ovarian fragment which also contained a Brenner nodule. In the same animal there was also a Wolffian cyst in the parametrium.

*Subserous fibroids.*—(*a*) A large fibroid located between the duodenum and colon was found in XI/18, of 44 months. The greater part of the tumour consists of collagenous tissue with interspersed nuclei. At some places in the periphery the number of cells is much greater and the nuclei larger. Patches of smooth muscle tissue, probably belonging to the muscle layers of the intestinal wall, were found, though rather at a distance from the latter. There was great abundance of lipoid tissue. Necrotic zones with leucocytes are located in the tumour at a variable distance from the abdominal wall. The tumour invading the muscular wall has destroyed at one place the intestinal mucosa. The whole mucosa is affected. There is superficial necrosis of the glands and the intestinal cavity is filled with fragments of glands, leucocytes and amorphous masses. Among the leucocytes and necrotic masses in the tumour, not far from the destroyed wall, fragments of glands also were present. (*b*) A small fibroid was present on the surface of the liver in VII/38, of 32½ months (Fig. 32). Structurally the tumour in this anoestrous animal was similar to oestrogen-induced fibroids after withdrawal of oestrogen (Lipschutz, 1950, p. 164). (*c*) A tiny subserous nodule was found on the surface of the spleen in the anoestrous animal VI/32. It was similar to the oestrogen-induced nodules of the same localization. (*d*) Fibroids originating in the meso of the tube, the so-called " apical " uterine fibroids, were present in VI/32 and VII/38. They were of small size.

*Tumour of the renal pelvis* (XIII/21, 44 months). The tumour is broadly attached to the wall of the renal pelvis and also fills the ureter (Fig. 37, 40, 41). Normal pelvic mucosa and submucosa are still present around the tumour, in a limited area. Layers of the mucosa are superimposed on a tissue composed of cells with dense nuclei (Fig. 38, 39). The question arises whether these cells have orginated from the mucosa or from the submucosa ; the first is the more probable. On the surface the cells sometimes form tubular structures which may be also cystic (Fig. 38) ; solid cords of cells or tubules with a stratified epithelium deeply penetrate the growth (Fig. 42, 43). In the deeper part of the submucosa the tissue is loose and contains many spindle shaped nuclei (Fig. 44, 45). The part of the tumour which fills the ureter is lobulated ; on the surface it consists of œdematous connective tissue with scattered clusters of cells (Fig. 40) ; at other places the tumour is solid (Fig. 41). In various parts of the ureter there are independent excrescences whose structure is similar to those of the pelvis.

*Condition of the suprarenals.*

In 4 animals the cortex offered an unusual picture. Islets of cortical cells resembling those of the zona fasciculata were present amidst medullary tissue (VII/39 and X/35) ; or there were nodules on the very surface of the gland (VI/32 and XII/23). We do not feel sure about how far this condition diverges from what may be found in normal animals.

### DISCUSSION.

The above description offers a picture of very manifold tumoural responses in a group of 17 animals necropsied 2 to 4 years after ovarian fragmentation or subtotal castration. As in former observations there were various types of atypical behaviour of uterine glands : adenomatous polyps filling the uterus ; an adenomatous polyp descending into the vagina ; uterine glands penetrating into the myometrium ; proliferated glands reaching the serosa and forming adeno-

TABLE I.—*Ten Female Guinea-pigs with "Ovarian Fragmentation," Necropsied $23\frac{1}{2}$ to $33\frac{1}{2}$ Months after Operation.*

| Number. | Weight.* | Duration of expt. in days. | Uterus. Wt., g. | Uterus. Microscopical condition. | Vagina and Mammary glands. | Ovary.† | Extragenital tumours. | Figs. |
|---|---|---|---|---|---|---|---|---|
| I/16 | 600—980 | 711 | 2·1 | Endometrium cylindrical. Cystic gland hyperplasia. Scattered groups of anaplastic glands. "Vascular" polyps; blood in uterine cavity. Glands in myometrium. | Oestrus. | F.III, C.l. Wolffian cysts Haemorrhagic follicles. | — | 11 |
| II/13 | 700—820 | 711 | 1·4 | Endometrium cylindrical. Vascular walls greatly thickened. | Metoestrus. | F.III. | — | |
| III/17 | 760—880 | 711 | 1·9 | Endometrium cylindrical. Glands in myometrium. Vascular walls greatly thickened. | Prooestrus. | F.III, C.l. Large Wolffian cyst. | — | |
| IV/14 | — | — | — | Adenocarcinoma, initial stage, see Table III. | | | | |
| V/15 | 810—860 | 711 | — | Endometrium cylindrical. Subserous adenofibromyoma and adenomyoma, both horns. Adenomyosis. Submucosa oedematous and very vascular; blood beneath endometrium and in uterine cavity. | Advanced metoestrus. | F.III, C.l. Large Wolffian cyst. | — | 5, 6, 8, 9 |
| | | | | | Mammary glands secreting. | — | — | |
| VI/32 | 590—350 | 945 | 1·0 | Endometrium and submucose-castrate. Cystic glandular hyperplasia, polyps; involution. Subserous adenofibroma, involution. "Apical" fibroid, 5 mm. | Anoestrus Mammary glands involvement | F.III Large Wolffian cyst. | Minute fibroid (?) on surface of spleen. | |
| VII/38 | 840—540 | 979 | 0·9 | Endometrium and submucous-castrate. Cystic glandular hyperplasia, large polyps; involution. "Apical" fibroid, 2 by 3 mm. | Anoestrus. | Only large Wolffian cysts and numerous Wolffian tubules | Adenomatous nodules (?) of the suprarenal cortex. Subserous desmoid of liver. | 32 |
| VIII/39 | 700—460 | 980 | 0·8 | Endometrium-castrate. | Anoestrus. | C.l., degeneration. Large Wolffian cyst. | Adenomatous nodules (?) of supra-renal cortex. | |
| IX/27 | — | — | — | Adenocarcinoma, multifocal, see Table III. | | | | |
| X/35 | 700—590 | 1012 | 0·7 | Endometrium and submucous-castrate. Cystic glandular hyperplasia, involution. | Anoestrus. | Degeneration ovarian tissue rete. | Adenomatous nodules (?) of suprarenal cortex. | |

* First figure—weight at operation; second figure—weight at necropsy.

† F.III—Graafian follicle; C.l.—corpus luteum.

TABLE II.—*Seven Female Guinea-pigs with "Ovarian Fragmentation," Necropsied $43\frac{1}{2}$ to $44\frac{1}{2}$ Months after Operation.*

| Number. | Weight, g. | Duration of expt. in days. | Uterus. Wt., g. | Uterus. Microscopical condition. | Vagina and Mammary glands. | Ovary. | Extragenital tumours. | Figs. |
|---|---|---|---|---|---|---|---|---|
| XI/18 | 660—690 | 1324 | 1·5 | Endometrium - castrate. Cystic gland hyperplasia, polyps, involution. Subserous adenofibroma, involution. | Anoestrus; solid epithel. cord in sclerotic wall of fornix. Mammary gland partly involution but partly with secretions. | Atretic follicles. Large Wolffian cyst. | Large desmoid between duodenum and colon. | 10 |
| XII/23 | 560—1030 | 1346 | 2·9 | Endometrium cylindrical. Metaplasia; glands transforming into almost squamous epithelium. Cystic gland hyperplasia. Subserous adenofibromyoma; cystic glands very vascular. | Oestrus, cornification. Mammary gland secreting. | F.III, C.l. Large Wolffian cysts "Brenner." | "Brenner" near the tube. Adenomatous nodules (?) of suprarenal cortex. | 4 3 7 |
| XIII/21 | 820—760 | 1346 | — | Endometrium cylindrical. Cystic glandular hyperplasia. Large polypus prolapse into vagina. | Metoestrus (?) Mammary gland secreting. | F.III, C.l. Large Wolffian cysts. Haemorrhagic follicles | Large Wolffian cysts. Fibrous nodules near the tube. Large tumour of renal pelvis. | 37–45 |
| XIV/10 | 500—1140 | 1351 | 2·3 | Endometrium cylindrical. Glands in myometrium. Glands partly anaplastic. | Metoestrus. Mammary gland secreting. | F.III, C.l. Large Wolffian cyst. Luteic cyst "Brenner" (?). Local hyperplasia of germinal epithelium. | — | |
| XV/7 | 580—890 | 1351 | 1·4 | Endometrium cylindrical. Glandular cysts. Glands in myometrium. | Oestrus. | C.l. Wolffian cyst. Haemorrhagic luteic cyst. | — | |
| XVI/8 | — | | — | Adenocarcinoma, unifocal; see Table III. | | | | |
| XVII/9 | — | | — | Adenocarcinoma, multifocal; see Table III. | | | | |

TABLE III.—*Four Female Guinea-pigs with "Ovarian Fragmentation" in which there was Uterine Adenocarcinoma.*

| Number. | Weight. | Duration of expt. in days. | Uterus. Wt. g. | Uterus. Microscopical condition. | Vagina and Mammary glands. | Ovary. | Extragenital tumours. | Figs. |
|---|---|---|---|---|---|---|---|---|
| IV/14 | 770—950 | 711 | 2·7 | Endometrium cylindrical. Large nodule of anaplastic glands: Initial stage of adenocarcinoma, unifocal. | Metoestrus. | F.II., C.l. Large Wolffian cysts. Luteic cyst. | | 17 |
| XV/I8 | 740—1130 | 1351 | 1·0 | Right: Endometrium cubical; some glandular cysts | — | — | — | |
| | | | | Left: Adenocarcinoma, unifocal. | Mammary glands secreting. | No active tissue Wolffian cyst. | — | 12–16 |
| IX/27 | 870—490 | 980 | 2·0 | Right: Endometrium cylindrical and cubical; mostly absent; in some places superficial necrosis. Submucosa very vascular; great abundance of glands. Many foci of adenocarcinoma. | Transition.* Mammary glands developed, but beginning involution. | F.III, C.l. Wolffian cysts "Brenner". | Extraovarian Wolffian cysts and tubules; highly vascular. Subserous ossification in lung. | 23 |
| | | | | Left: Adenocarcinoma, lobulated, distending the uterus. Solid cords of cells originating from submucosa. Various large foci of adenocarcinoma in uterus beneath the principal focus. Anaplastic glands, but no nodules in uterus above principal focus. | — | — | — | 18–22, 24 |
| XVII/9 | 750—1160 | 1351 | 12·7 | Right: Adenocarcinoma, multifocal. Solid masses of cells, originating from submucosa covered by fibrous capsule, with no endometrium; loose and oedematous fibrous tissue; necrosis. Remaining endometrium cylindrical; glands with high epithelium. Upper part of right uterus: Endometrium cylindrical. Glands in myometrium. Small nodules of anaplastic glands. Left: Endometrium cylindrical. Submucosa highly vascular. Glands in myometrium. Some groups of anaplastic glands. | Oestrus. | F.III, C.l. Cysts of the rete "Brenner". Local hyperplasia of germinal epithelium. | — | 25, 29 1, 2 26, 30, 31 27, 28 |

* Transition pregnancy to oestrus, or *vice versa*; or action of oestrogen incompletely antagonized by progesterone.

### EXPLANATION OF PLATES.

FIG. 1.—Local increase of germinal epithelium, XVII/9. × 70.
FIG. 2.—The same, XVII/9. × 300.
FIG. 3.—Nodule of Brenner type in the ovary. Ovum on the top, to the right, XII/23. × 70.
FIG. 4.—Extra-ovarian nodule of Brenner type. Tube on the left, XII/23. × 70.
FIG. 5.—Subserous adenofibromyoma. Many glands enlarged. Blood beneath the endometrium and in the uterine cavity, V/15. × 15.
FIG. 6.—Subserous adenomyoma, V/15. × 23.
FIG. 7.—Subserous adenofibromyoma with highly enlarged glands, XII/23. × 15.
FIG. 8.—Glands of adenofibromyoma of Fig. 5, V/15. × 300.
FIG. 9.—Uterine glands of the same animal. Note the difference between Fig. 8 and 9, V/15. × 300.
FIG. 10.—Nodule attached to the uterine wall. Various cysts with low epithelium, surrounded by fibrous tissue rich in cells. Small fibrous nodule poor in nuclei in the lower half of the nodule, XI/18. × 15.
FIG. 11.—Uterine polyp consisting principally of blood vessels. Endometrium cylindrical. At different places stratification (?) of endometrium, I/16. × 45.
FIG. 12.—Adenocarcinoma without invasion of uterine wall, XVI/8. × 15.
FIG. 13.—Detail of Fig. 12. Some normal glands on the top to the right, beneath endometrium. Note the deformation of glands of adenocarcinoma, XVI/8. × 70.
FIG. 14.—Part of adenocarcinoma where deformation of glands is more pronounced than in Fig. 13, XVI/8. × 70.
FIG. 15.—Glands of adenocarcinoma of the same animal. Solid epithelial outgrowths (?) of various glands, XVI/8. × 300.
FIG. 16.—Seemingly normal glands to which attention has already been attracted in Fig. 13. Compare with glands of adenocarcinoma, XVI/8. × 300.
FIG. 17.—Nodule of anaplastic glands beneath flattened endometrium. The difference between these glands and seemingly normal glands in the upper part of the figure can be seen already at low magnification, IV/4. × 15.
FIG. 18.—Left uterine horn locally distended by growth, IX/27. Normal size.
FIG. 19.—Growth seen in Fig. 18, IX/27. × 3.
FIG. 20.—Highly deformed glands of the growth seen in Fig. 19, IX/27. × 70.
FIG. 21.—Large blood vessel in septum separating lobules of the growth, IX/27. × 70.
FIG. 22.—Solid mass of cells amidst glands of adenocarcinoma. The cells belong most probably to the submucosa. On the top-fibrous tissue, IX/27. × 70.
FIG. 23.—Nodules of adenocarcinoma in the opposite (right) horn of the same animal, IX/27. × 15.
FIG. 24.—Nodule of adenocarcinoma in the individual cervix of one of the horns. Note that the normal cervix is lined with mucified cells, IX/27. × 15.
FIG. 25.—Large growth in the right horn distending it greatly, XVII/9. Normal size.
FIG. 26.—Distended uterine wall covering a nodule of adenocarcinoma. On the top to the left—nodule of enlarged cells of the submucosa with many blood vessels. Beneath the adenocarcinomatous part—large masses of cells of the submucosa. In the centre and to the left, possibly necrobiotic changes, XVII/9. × 15.
FIG. 27.—Cylindrical endometrium of right horn. Beneath the tunica—part of myometrium, XVII/9. × 300.
FIG. 28.—Large gland in tunica beneath endometrium of right horn, XVII/9. × 300.
FIG. 29.—Glands of adenocarcinoma of right horn, XVII/9. Compare with Fig. 27 and 28. × 300.
FIG. 30.—Solid nodule of cells of the submucosa reaching the surface of the growth. To the left—fibrous tissue, cellular and non-cellular, XVII/9. × 15.
FIG. 31.—Solid nodule of large cells probably originating from submucosa. The nodule is covered by fibrous tissue probably also belonging to the submucosa, XVII/9. × 300.
FIG. 32.—Desmoid on the surface of the liver, VII/38. × 70.
FIG. 33.—Endometrium of animal with subcutaneously implanted pellet containing 5 per cent of oestradiol, 16 months, CXXIII.51. × 300.
FIG. 34.—Anaplastic uterine glands, same animal as Fig. 33, CXXIII.51. × 300.
FIG. 35.—Anaplastic gland with very doubtful picture of solid outgrowth, same animal as Fig. 33 and 34, CXXIII.51. × 300.
FIG. 36.—Uterine gland, same animal as Fig. 33 to 35. Part of the gland shows cells similar to those of the endometrium (Fig. 33) whereas another part of the same gland is constituted of anaplastic glands similar to those in Fig. 34 and 35. × 300.
FIG. 37.—Tumour of renal pelvis. Ureter greatly distended by the tumour, XIII/21. × 1·4.
FIG. 38.—Epithelium of the pelvis superimposed on tissue of cells with dense nuclei. Note tubular structure and cystic dilatation on the surface, XIII/21. × 70.
FIG. 39.—Surface epithelium and layers beneath reaching considerable thickness, XIII/21. × 70.
FIG. 40.—Part of the tumour in the ureter. To the left, oedematous connective tissue with clusters of large cells, XIII/21. × 15.
FIG. 41.—Solid part of the tumour in the ureter, XIII/21. × 300.
FIG. 42.—Tubule with stratified epithelium beneath the surface, XIII/21. × 70.
FIG. 43.—Structure similar to that of Fig. 42, but less delimited, XIII/21. × 300.
FIG. 44 and 45.—Deeper part of the tumour with many spindle shaped nuclei, XIII/21. × 300.

1
2
3
4
5
6
7

§

Bruzzone and Lipschutz.

Bruzzone and Lipschutz.

21

22

24

23

25

26

Bruzzone and Lipschutz.

27

28

29

32

30

31

Bruzzone and Lipschutz.

Bruzzone and Lipschutz.

42

43

44

45

fibromyoma or adenomyoma. Similar responses on the part of the uterine glands can be elicited in the guinea-pig with the prolonged administration of oestrogens, and it is but reasonable to suppose that they were due, in our group of animals, to protracted phases of oestrogenic action not duly interfered with by progesterone.

Two new aspects referring to the glands penetrating into the myometrium, aspects not yet dealt with in former work, deserve special mention. The first one is the fact that the glands found in the myometrium or those of the adenofibromyomata are often structurally different from normal uterine glands. The penetrating glands have undergone structural changes. The dynamics of these changes are wholly unknown to us ; but it would be unreasonable to ascribe them to the influence of the new " ambiental " conditions into which the penetrating glands are entering, since we have seen structural changes of glandular cells so fundamental as those summarized as anaplasia may take place without penetration.

A second aspect of interest is the probability that the adenofibromyoma elicited by the protracted action of oestrogen is reversible. There were 2 animals (VI/32 and XI/18) with an endometrium of the castrate type, and in these animals bodies attached to the uterus were found which were most probably involuted adenofibromyomata (Fig. 10). This, indeed, is only a tentative explanation. But in its favour was the castrate condition of the uterine and vaginal epithelium in both these animals, and the involution of the mammary glands. The fibrous mesenteric tumour in one of these animals (XI/18) was of the desmoid type, and fibroblasts characteristic of the growing oestrogen-induced fibroid (Lipschutz, 1950, Fig. 10) were absent.

Of considerable interest is the new and unexpected finding of uterine adenocarcinoma which was in full development in 3 animals, and in an initial stage in one animal. Like the adenomatous polyp, this adenocarcinoma has no tendency to penetrate into the myometrium. Metaplasia of the endometrium elicited by the prolonged action of oestrogen has been described in the guinea-pig (Lipschutz 1950, Fig. 62) and other species by various authorities ; it has been seen also in guinea-pigs with ovarian fragmentation (Lipschutz, 1937). But never before have we seen in experiments with ovarian fragmentation an anaplastic condition similar to that of Fig. 15 and 29 of the present series. Scattered groups of anaplastic glands were found also in 2 other animals (I/16 and XIV/10), though the anaplasia was not of the same degree as in XVI/8, IX/27 and XVII/9.

We have now to discuss the question whether the adenocarcinoma was due to oestrogen acting in the body of our animals with ovarian fragmentation. An anaplastic condition of the glands (Fig. 33–36), similar to that of adenocarcinoma in the present work, a condition so different from that of normal uterine glands subject to the action of oestrogen, has been observed, though only once, in a series of castrated animals with the absorption, from a subcutaneously implanted pellet, of small quantities of oestrogen during more than 16 months (experiments in collaboration with A. Riesco ; Lipschutz, 1950, p. 84).

We must now discuss the condition of the ovarian remnant in the 4 animals with uterine adenocarcinoma including the initial stage. The findings were rather conflicting. Large Graafian follicles were present in XVII/9, but in 2 other animals they were small or atretic (IV/4, IX/27) and no active ovarian tissue was found in XVI/8. Corpora lutea in IX/27 and XVII/9 were in a state of degeneration, but a luteic cyst was present in XVII/9. However, in none of these animals was the uterine weight that of a castrate ; the vaginal mucosa was in oestrus or metoestrus ; in XVI/8 the mammary gland was in a state of secretion. It is thus evident that in the 4 mentioned animals there was, in the course of the experiment and till lately, secretion of oestrogen and probably also of progesterone ; but nothing can be said as to the quantitative and timing conditions of their secretion. However, the fact that in the present series luteic cysts or haemorrhagic follicles were found in no less than 5 or 6 animals is sufficient to warrant the assumption that there was abnormal hypophysial activity also in the 4 cases with uterine adenocarcinoma as it generally occurs in ovarian fragmentation in the guinea-pig (see discussion in Iglesias, Mardones and Lipschutz, 1935*b*). An abnormal hypophyseal activity is also evidenced in XVII/9 by the extraordinary condition of the germinal epithelium (see Section " Ovarian Fragment "), and likewise by the masculinization of the genital region in 3 animals (IV/4, XVI/8 and XVII/9) ; there was growth of the clitoris and of horny styles as present in the male (similar findings in Lipschutz, 1938). No more than that can be said at the moment with reference to the hormonal dynamics of the adenocarcinomatous growth in the uteri of our animals.

There is another very relevant question. Our experimental animals were all aged animals. At necropsy none was less than 3 years old ; the 3 animals with highly developed uterine adenocarcinoma (XVI/8, IX/27 and XVII/9) were certainly 4 to 5 years old. One may ask whether a similar tumoural growth may occur in non-operated old guinea-pigs. But even should it be so this would not be contrary to the assumption that a hormonal imbalance is at the root of this kind of uterine tumourigenesis.

As already insisted upon, superficial uterine adenocarcinoma as in our 4 animals, without penetration into the myometrium, has not been seen in our former work with ovarian fragmentation or in that of other authorities who used the same method. There is the possibility that genetic conditions are responsible for the variable results.

When dealing with uterine adenocarcinoma in our guinea-pigs (Table III) it is of interest to note the fact that a superficial layer of adenocarcinoma with little penetration into the muscle is not unusual in women (Willis, 1948 ; p. 536) ; multifocal origin as in IX/27 and XVII/9 probably also occurs in women.

The ovarian nodule of the Brenner type found in XII/23, IX/27 and XVII/9 is identical with that we have described in intrasplenic ovarian grafts in the same species (Iglesias, Mardones, Bruzzone and Lipschutz, 1953 ; Iglesias, Mardones and Lipschutz, 1953*a*). A similar tumoural response of ovarian structures has already been seen in former work with ovarian fragmentation (Lipschutz, 1938 ; 1950, p. 223).

A quite unexpected finding was the large tumour of the renal pelvis and the excrescences of the mucosa of the ureter. The structure of this growth reminds one of the papillary carcinoma of the renal pelvis in man as pictured by Anderson (1948, p. 662). But we do not dare yet to classify the tumour found in our present work. Carcino-sarcoma may be the appropriate name ; it seems very likely that it originated from the mucosa of the pelvis. We may remember that proliferation of the epithelium of the urinary tract subject to the action of oestrogen is a well known phenomenon and has more recently attracted attention also in clinical work (Del Castillo and Argonz, 1953). In the same animal there was also a huge uterine polyp descending into the vagina. The ovarian remnant contained a haemorrhagic follicle.

SUMMARY.

Guinea-pigs with ovarian fragmentation, or subtotal castration, were necropsied about 2 to 4 years after operation.

The condition of the ovarian fragment gave full evidence that an ovarian-hypophyseal hormonal imbalance had taken place. Among 17 animals there were 2 with haemorrhagic follicles and 3 with luteic cysts, one of which was haemorrhagic. In 2 animals there was localized hyperplasia of the germinal epithelium.

Most of the ovarian fragments contained Wolffian cysts ; in 3 animals the fragment contained small tumours of the Brenner type.

In several animals the fragment contained an abundance of follicular clusters originating from small atretic follicles.

As in former experiments with ovarian fragmentation cystic glandular hyperplasia occurred, with considerable vascularization of the tunica. In several animals polyps of the endometrium filled the uterine cavity and in 1 animal the polyp prolapsed into the vagina. Glands penetrated deeply into the myometrium.

In 2 animals large subserous adenofibromyomata were found. In 2 other animals there were subserous nodules which were seemingly involuted adenofibromyomata.

Endometrial adenocarcinoma of the " superficial " type (Willis, 1948) was found in 3 animals necropsied 33 to 45 months after operation. The adenocarcinomatous nodules were of variable size, reaching in 1 animal a diameter of more than 2 cm., and consisted of anaplastic glands. The latter differed from normal glands in three respects : (1) the cells were poorer in protoplasm and the nuclei were denser ; (2) the anaplastic glands were very frequently of irregular shape ; (3) these glands showed seemingly solid epithelial outgrowths. The adenocarcinoma was unifocal in 1 case, and multifocal in the 2 remaining cases . A small nodule of anaplastic glands was found in a fourth animal about 2 years after operation, and scattered groups of similar anaplastic glands were present in 2 other animals. However, in none of the last 3 animals was the anaplasia of the same degree as in the 3 cases of adenocarcinoma mentioned above.

Various extra-uterine tumours were also found : a nodule of the Brenner type near the tube ; a large fibroid, or desmoid, between the duodenum and colon ; a small fibroid, or desmoid, on the surface of the liver ; so-called " apical " uterine fibroids originating in the meso of the tube. A large tumour of the renal pelvis, probably a carcino-sarcoma originating from the mucosa, deserves special notice.

The question is discussed how far tumourigenesis due to the hormonal imbalance in experiments with ovarian fragmentation has been enhanced in these experiments of such a long duration by the advanced age of the animals.

Our thanks are due to Dr. R. Barahona, Professor of Pathology at Universidad Católica de Chile, for helpful and generous advice in microscopical diagnosis. Thanks are due also to the technical staff of the Institute including the Photographer and Secretary.

REFERENCES.

ANDERSON, W. A. D., Ed.—(1948) ' Pathology.' St. Louis (Mosby).

BRUZZONE, S.—(1950) *Rev. Med. Aliment., Santiago,* **9**, 11.

Burch, J. C., Wolfe, M. M., and Cunningham, R. G.—(1932) *Endocrinology*, **16**, 541.
Del Castillo, E. B., and Argonz, J.—(1953) *Acta physiol. Lat.-Amer., Buenos Aires*, **3**, 85.
Fels, E.—(1942) *Rev. Soc. argent. Biol.*, **18**, 286.—(1947) *Ibid.*, **23**, 10.
Iglesias, R., Mardones, E., and Lipschutz, A.—(1953*a*) *Brit. J. Cancer*, **7**, 214.—(1953*b*) *Ibid.*, **7**, 221.
*Idem*, Mardones, E., Bruzzone, S., and Lipschutz, A.—(1953) *Arch. Anat. micr. Morph. exp.*, **42**, 3.
Lipschutz, A.—(1931) *Endokrinologie*, **29**, 258.—(1936*a*) *C. R. Acad. Sci., Paris*, **203**, 1025.—(1936*b*) *Arch. Biol., Paris*, **47**, 181.—(1936*c*) *C. R. Soc. Biol., Paris*, **123**, 545.—(1937) *Gynéc. et Obstét.*, **36**, 407, 481.—(1938) *Ibid.*, **37**, 17.—(1950) 'Steroid Hormones and Tumours.' Baltimore (Williams & Wilkins).
*Idem* and Osnowikoff, B.—(1932) *C. R. Soc. Biol. Paris*, **111**, 350.
*Idem* and Voss, H. E.—(1925) *Brit. J. exp. Biol.*, **3**, 35.
Morató, J.—(1941) *Endocrinology*, **29**, 619.
Nadel, E. M.—(1949) *J. nat. Cancer Inst.*, **9**, 271.
Navori, C. A., Fugo, N. W., and Davis, M. E.—(1952) *Proc. Soc. exp. Biol. N.Y.*, **81**, 649.
Ponse, K., and Dovaz, R.—(1950) *Ann. Endocr., Paris*, **11**, 426.—(1951) *Ibid.*, **12**, 150.
Willis, R. A.—(1948) 'Pathology of Tumours.' London (Butterworth).